# Lateral and Medial Elbow Tendinopathy and Previous Injuries to Adjacent Joints: A Multicenter Observational Study

**DOI:** 10.3390/healthcare12171758

**Published:** 2024-09-03

**Authors:** Maria Jesus Vinolo-Gil, Ismael García-Campanario, María José Estebanez-Pérez, Manuel Rodríguez-Huguet, Marta Linares-Gago, Francisco Javier Martin-Vega

**Affiliations:** 1 Department of Nursing and Physiotherapy, Faculty of Nursing and Physiotherapy, University of Cadiz, 11009 Cadiz, Spain; manuel.rodriguez@uca.es (M.R.-H.); javier.martin@uca.es (F.J.M.-V.); 2 Rehabilitation Clinical Management Unit, Interlevels-Intercenters Hospital Puerta del Mar, Hospital Puerto Real, Cadiz Bay-La Janda Health District, 11006 Cadiz, Spain; marta.linaresgago@gmail.com; 3 Research Unit, Biomedical Research and Innovation Institute of Cadiz (INiBICA), Puerta del Mar University Hospital, University of Cadiz, 11009 Cadiz, Spain; 4 Group PAIDI UCA CTS391, Faculty of Medicine, University of Cadiz, 11003 Cadiz, Spain; ismael.garcia@uca.es; 5 Department of Physiotherapy, Faculty of Health Science, University of Malaga, 29071 Malaga, Spain; mariajoseestebanezperez@uma.es; 6 Department of Physiotherapy, Faculty of Health Science, University of Granada, 18071 Granada, Spain

**Keywords:** epicondylitis, epicondylosis, epicondylalgia, elbow tendinopathy, observational study, survey

## Abstract

Background: Lateral and medial elbow tendinopathies are common soft tissue disorders affecting 1–3% of the general population, causing significant pain and functional impairment in the elbow and upper limb. While often associated with overuse and repetitive strain, their exact etiology, including potential associations with prior injuries in adjacent joints, remains unclear. This preliminary study aims to explore the distribution of lateral and medial elbow tendinopathies and investigate the occurrence of previous lesions in adjacent joints among diagnosed individuals, providing foundational insights for future research. Methods: A multicenter cross-sectional observational study was conducted involving 90 subjects diagnosed with lateral and/or medial elbow tendinopathy. The data collection occurred during the initial consultations, including demographic information, clinical assessments, and history of prior injuries in adjacent joints. Results: Among the sample, 44.4% reported prior injuries to adjacent joints in the affected upper limb, with 45.6% of these injuries identified as musculotendinous in nature. The analysis also showed that the type of elbow tendinopathy was significantly associated with sex (*p* = 0.01) and occupational origin (*p* = 0.022). Conclusions: While a notable percentage of the subjects reported prior musculoskeletal injuries in the same limb, the study’s geographic limitations and reliance on self-reported data introduce potential recall bias. These preliminary findings suggest a possible relationship between prior adjacent joint injuries and elbow tendinopathy. Further research with larger sample sizes and more rigorous study design is needed to confirm these observations and explore the underlying mechanisms.

## 1. Introduction

Elbow tendinopathies, encompassing conditions like lateral and medial elbow tendinopathies (LETs and METs), are marked by pain and limited function in the upper limb [1,2]. Previous terms like “tennis elbow” and “golfer’s elbow” inaccurately describe these conditions and their underlying causes [3]. Histological studies indicate that a phase of inflammation occurs, followed by tendon degeneration, supporting the term “tendinosis” for these conditions [4,5]. LETs and METs significantly contribute to elbow pain, affecting approximately 3.4 individuals per 1000, with peak incidences typically seen between the ages of 40–49 in men and 50–59 in women [6,7].

Treatment is generally conservative, including rest from physical and occupational activities associated with an exacerbation of symptoms, the use of non-steroidal anti-inflammatory drugs (NSAIDs) for pain relief, and physiotherapy focusing on progressive strengthening, endurance, and stretching of the forearm muscle. Corticosteroid infiltrations can be considered as an additional therapeutic option [8], although surgical treatment is sometimes necessary in the most stubborn cases [7].

Although Ikonen et al. [1] describe a high probability of spontaneous resolution, the chronicity of this injury, together with the intensity of the pain, which sometimes does not correspond to the degree of the disability it produces, has a psychogenic effect on the patient, affecting their quality of life [9,10,11,12]. Moreover, recent research emphasizes the neurogenic aspects of lateral elbow tendinopathy, highlighting complex interactions involving peripheral sensitization and neuropathy [13,14]. These findings underscore the importance of considering these neurogenic aspects in the assessment and treatment of lateral elbow tendinopathy. An integrative model for lateral epicondylalgia proposed in the scientific literature suggests that the condition may arise from a combination of biomechanical, neurological, and psychosocial factors. This model helps to illustrate that the condition may develop in response to various activities involving repetitive forearm movements and overloading, which are not limited solely to tennis [15].

The occurrence of this overuse injury [16] is commonly associated with occupational activities [1,6,17,18] and sports, particularly racket sports [19,20], though it can also manifest in other athletic pursuits [21]. Understanding the etiology of these tendinopathies is crucial for effective prevention efforts [18].

Increased stress on the forearm and forearm rotation, along with overuse of the musculature, have been identified as primary causes [17]. These factors can lead to issues such as blood circulation problems, aging, and decreased flexibility [9]. Specifically, LET is attributed to eccentric loading at the origin of the common extensor tendon, resulting in tendinosis and inflammation of the extensor carpi radialis brevis (ECRB). On the other hand, MET is associated with eccentric loading of the flexor–pronator group at the medial epicondyle [22].

Furthermore, the studies by Alfredson et al. [23] and Connel et al. [24] showed no inflammatory signs in the injured tissue, but instead the degeneration of collagen and proliferation of fibroblastic cells. There are different theories explaining the occurrence of such a process, termed “tendinosis” or “angiofibroblastic hyperplasia” [25], which occur as a consequence of incomplete tendon repair. These theories suggest that our body may be unable to fully repair the injured tissue due to the intensity of the destruction exceeding the regeneration process [26]. Alonso et al. [27] described the existence of other concomitant lesions on the shoulders, elbows, forearms, and wrists, calling it a “mesenchymal syndrome”. In some clinical scenarios, overuse and repetitive trauma are not the sole etiological factors; these patients also present with multiple symptoms of tendinosis, often affecting both sides, including the shoulders and the medial and lateral epicondyles, as well as carpal tunnel syndrome. By focusing on the elbow, our study aims to investigate these injuries within the context of tendon disorders, which are the primary manifestations of this syndrome. The similarity in the lesioning process across different body parts suggests a potential genetic predisposition in the tendon structure [26]. Haahr et al. [28] observed such concurrent lesions even preceding those in the elbow in their case-referent study involving 267 new cases of tendon pathology and 388 participants from the general population in Denmark. These authors identified significant physical and psychosocial risk factors associated with the development of tendinopathies.

Given these findings, it is crucial to investigate potential predisposing factors for tendinopathy, including the presence of previous injuries in adjacent joints. The aims of this multicenter study are twofold: to examine the manifestation of lateral and medial elbow tendinopathies among individuals diagnosed with these conditions, and to explore any potential associations between these types of tendinopathies and previous injuries in adjacent joints. Specifically, we seek to identify whether prior injuries in adjacent joints are linked to the development or progression of elbow tendinopathy.

## 2. Materials and Methods

### 2.1. Subjects

The study sample consisted of 90 patients diagnosed with epicondylosis treated at the Rehabilitation and Physiotherapy Services of three Primary Health Care centers of the Andalusian Health Service, located in Puerto Real, Medina-Sidonia, and Chiclana de la Frontera, all belonging to the province of Cadiz (Spain).

### 2.2. Sample Determination

For this observational study, the sample size was determined using a convenience sampling method, based on the availability of patients at the participating centers and the aim to capture a diverse range of cases of both lateral and medial elbow tendinopathies. Our objective was to include a sufficient number of participants to explore significant associations between elbow tendinopathies and previous injuries in adjacent joints. Given the continuous recruitment process over twelve months, we implemented follow-up measures to ensure robust data collection.

### 2.3. Study Procedure

This is a cross-sectional, multicenter observational study conducted on subjects diagnosed with elbow tendinopathy (medial and/or lateral). The recruitment period for this study was twelve months (from November 2021 to November 2022). This investigation was submitted to and approved by the Research Ethics Committee of the province of Cadiz (Spain), with registration number 27/20 and protocol code 0623-n-20.

All ethical guidelines and principles included in the Declaration of Helsinki were followed, as well as current legislation on data protection.

### 2.4. Data Collection

The collection of the necessary data was carried out in a single consultation and was performed by the Specialist Physician in Physical Medicine and Rehabilitation, after carrying out the diagnosis and subsequent acceptance and inclusion of the subject in the study group. Specifically, the diagnosis was confirmed by the following: localization of the pain at the elbow, positive contraction maneuvers of the wrist and finger extensors for lateral epicondylalgia (Cozen’s test) [29], and contraction maneuvers of the wrist and finger flexors for medial epicondylalgia [30], as well as ultrasound to assess the integrity of the tendons and to rule out the presence of a tear [30,31,32]. Additionally, neurogenic factors were considered by evaluating the presence of neuropathic pain features and sensitization symptoms [33].

The rehabilitation doctors of the clinical management unit, who are responsible for the three study centers, hold annual clinical sessions on musculoskeletal pathologies to update and unify diagnostic criteria. Within the unit, we have two doctors dedicated exclusively to musculoskeletal pathologies of the upper limb, which ensures a high level of specialization and consistency in diagnoses.

The data consisted of a survey carried out using a Google Drive form, which was completed by the Rehabilitation Doctors with their mobile phones using a QR code.

In terms of inclusion criteria, adult subjects over 18 years of age were eligible for the study if they had a clinical diagnosis of medial and/or lateral tendinopathy made by the Rehabilitation Physicians involved in this study. Participants needed to provide informed consent to be included in this study. Furthermore, individuals exhibiting neuropathic pain features and sensitization symptoms as part of their diagnostic evaluation were also included, in order to address potential neurogenic factors. Regarding exclusion criteria, patients were not included if they had significant psychological or neurological alterations that could hinder the collection of necessary information for the research. Additionally, those involved in legal disputes that might be affected by their participation in this study were excluded. Patients with confirmed tendon tears were also excluded, as these conditions could potentially interfere with the study outcomes. Moreover, individuals with primary neurogenic pain conditions unrelated to elbow tendinopathies were excluded to ensure that our study remained focused on the specific pathology under investigation.

### 2.5. Outcome Variables

Data collection consisted of three main components: socio-demographic characteristics (sex and age), clinical assessments, and work and leisure details.

Regarding clinical assessments, three injury types were evaluated: medial epicondylosis, lateral epicondylosis, and combined lateral/medial epicondylosis. The affected upper limb was classified as right, left, or bilateral. For bilateral cases, participants indicated the initial site of symptom appearance (right arm, left arm, or uncertain), identified their dominant arm, and noted if it was their first instance of such pathology. Other collected data included the approximate duration of onset in days, potential causes of injury, and prior injuries to adjacent joints in the affected upper limb. If applicable, participants specified whether injuries occurred in the shoulder or hand–wrist joint complex, and categorized them as muscle–tendon, osteo–articular, or osteo–tendon injuries. Furthermore, participants were queried about injuries in adjacent joints of the unaffected upper limb.

Concerning work and leisure variables, participants provided information on their employment status at the time of data collection, profession, and whether their occupation involved manual handling. Lastly, participants indicated if they engaged in any racket sports.

### 2.6. Data Analysis

A descriptive analysis of all the variables included in this study was presented at the global level and by type of epicondylosis. For qualitative variables, relative and absolute frequencies have been presented. For quantitative variables, summary statistics (mean, median, SD, minimum, and maximum) were used. For all the objectives of this study in which the objective was to relate two qualitative variables, the non-parametric Chi-square test was used and, if it was necessary, Fisher’s test was calculated (in cases where the absolute frequency of a level is less than 5 observations). To compare quantitative variables with qualitative variables that included three categories of classification, the ANOVA test was used. Missing values were considered as those that were not completed, as well as levels of qualitative variables that were reported as ‘do not recall’. All analyses were carried out with the free software R (V.4.1.1) and the level of significance for all hypothesis testing was determined at 0.05.

## 3. Results

The participants’ ages ranged from 19 to 80 years with the mean age being 44 years (SD = 12.1). There was an almost equal participation rate in this survey, with 51.1% women compared to 48.9% men. In this sample, 87.8% of the participants were predominantly right-handed.

Regarding the most prevalent area of injury location, LETs represented 74.4%, compared to METs with 13.3%. “Bilateral epicondylosis”, referring to the occurrence of epicondylosis in both the right and left elbow in the same individual, represented 12.2%. The right upper limb is the most affected, which accounted for 64.4%, compared to 27.8% on the left, or 7.8% in the case of a bilateral injury. If the respondent stated that the injury was bilateral, 11.1% the respondents considered the injury to have started on the right side of the body.

Nearly nine out of ten participants (88.9%) reported experiencing epicondylosis for the first time. Concerning the time with the symptomatology, 14.4% of the sample had been living with it for more than 1 year. The possible cause of the onset of the injury was work-related in 64.4% of cases. There was a percentage of the population (11.1%) that did not know how to recognize the causes of the origin of the injury.

Concerning previous injuries to adjacent joints of the affected upper limb, 44.4% reported such injuries, predominantly affecting the shoulder (27.8%). These injuries were primarily musculotendinous in nature (45.6%). In the unaffected upper limb, 17.8% reported prior injuries to an adjacent joint.

With regard to the occupation of the participants, 68.9% were active workers, 20% were unemployed, and 4.4% were retired. A large number of the subjects acknowledged that their profession requires them to do manual work (80%). Finally, in terms of leisure and recreational activities such as racket sports, 14.4% of the sample recognized that they engage in these activities.

The variable type of the injury (lateral, medial, or bilateral) showed significant differences by both sex (*p* = 0.01) and occupational origin (*p* = 0.022). However, no association was found with the other variables such as the injured upper limb, dominant limb or previous injury (Table 1).

In Table 2, a statistically significant association was found between the type of the affected limb and the dominant limb, with a *p*-value of 0.014. This suggests that the affected limb is associated with the dominant limb in our sample. However, no significant associations were found between the type of the affected limb and the other variables studied, such as sex, cause of injury, previous existence of injury in the non-affected limb, manual load by occupation, time of evolution, or age.

## 4. Discussion

Our study aimed to investigate the distribution and characteristics of lateral and medial elbow tendinopathies among individuals diagnosed with this condition, as well as their potential association with prior injuries in adjacent joints. Despite a high percentage of participants reporting previous issues in the affected upper limb, we did not observe a statistically significant relationship between these prior conditions and the occurrence of epicondylosis.

Regarding sex, some studies describe a higher number of cases in females [6,34]. However, in relation to lateral epicondylosis, this study has not observed statistically significant differences in relation to sex. These results are more in line with the studies by Yaka et al. and Keijsers et al. [35,36], although, in cases of medial and bilateral epicondylosis, there is a predominance of the female sex over the male one, coinciding with the study by Konarski et al. [37]. It should be mentioned, with regard to age, that despite the wide age range of the participants (19 to 80 years), the most prevalent age of 44 years falls within the ranges established in other studies [6,36,38]. This is in contrast to Degen et al. [7], who found a high incidence in people older than 65 years. Regarding the location of the injury, the right upper limb is the most affected, coinciding with the dominant upper limb; this is in agreement with Sghir et al. [6]. This observation underscores the higher incidence of epicondylosis in the dominant arm, which can be attributed to both occupational and recreational activities involving repetitive forearm movements [39,40]. The increased manual control and frequent use of the dominant limb further support the prevalence of lesions appearing in this limb [9,41]. Even in a significant number of the cases of bilaterality, the patient recognized that the injury began in the dominant upper limb. LET is the most frequent location in this study, occurring in 13.3% of patients. This difference is in agreement with the study by Konarski et al. [37]. The large percentage of individuals in this study who defined the cause of their injury as occupational in nature, who mostly had manual labor jobs, is in line with two previous studies [17,18] and in contrast with the study by Sghir et al. [6], where the majority of their participants had professional jobs of an administrative nature. However, there was a percentage of the patients (14.4%) who described the cause of their injury as being of a sporting nature, mainly racquet sports or other leisure activities. This result is in line with other studies [11,19,20,37,42].

It is interesting to note that 44.4% of the individuals in this study had previously suffered some type of musculoskeletal injury to the same injured upper limb, mainly at the level of the shoulder joint. This observation is consistent with the study of Descatha et al. [43]. The occurrence of previous injuries in adjacent joints could lead to muscle weakness in these areas and affect the movement of the arm as a whole, from a biomechanical point of view, facilitating the development of elbow injuries [44]. Irrespective of possible alterations at the tissue level, there seems to be a possible association between the biomechanical exposure of the elbow and wrist joint complex and this type of injury, as shown in a meta-analysis by Descatha et al. [43]. Although in this study a similar percentage of the patients (46.5%) had suffered similar adjacent injuries in the unaffected upper limb, the greater performance of manual tasks with the dominant upper limb may account for the lower predisposition to develop epicondylosis in the non-dominant upper limb [9,41].

With regard to the time of evolution of the lesion, a large majority (89.9%) described suffering from this type of condition for the first time and only 14.4% of the participants in this study presented with a clinical picture of more than one year, with these results coinciding with the study carried out by Ikonen et al. [1]. This could be due to the self-limiting nature of the clinical picture of this pathology [1,45,46].

Considering the significance of both pain intensity and functional limitations, which are crucial aspects that influence both the clinical management and quality of life of people with tendon injuries, future research efforts should incorporate validated assessment tools to quantify pain severity, assess functional restrictions in daily activities, and explore how these factors evolve over time. Recent literature suggests the utility of multimodal sensorimotor assessments of the hand and forearm, which offer a comprehensive view of sensorimotor function beyond conventional measures [15,47].

Existing literature highlights the significant influence of previous injuries on the experience and development of symptoms in the elbow region and adjacent areas. For example, studies have shown that individuals with a history of distal radial fracture show an expanded distribution of pain referred to the wrist. Specifically, pressure-induced pain in the extensor carpi radialis longus (ECRL) muscle frequently radiates to the distal forearm in these individuals [48]. Furthermore, lateral epicondylosis has been identified as a significant risk factor for rotator cuff disease, further highlighting the interconnectedness of musculoskeletal conditions in the upper extremity [49]. This evidence underscores the importance of considering the impact of past injuries when assessing musculoskeletal disorders in the upper extremity.

Finally, it is important to highlight the potential role of metabolic factors in the development of tendon injuries, which should be considered in future research. Studies have shown significant associations between metabolic conditions and the risk of tendon injuries. For instance, Skovgaard et al. [50] found that “hypercholesterolemia (total cholesterol >5 mmol/L) increased the risk of tendon injury in the upper extremities by approximately 1.5 times, and individuals with metabolic syndrome had approximately 2.5 times higher risk of tendon injury in both upper and lower extremities”. Additionally, in studies conducted on other tendons of the upper extremities, such as the rotator cuff, individuals with diabetes exhibit tendon degeneration resulting from the formation of advanced glycation end products and subsequent cross-linking within collagen fibers, which can impair mechanical function [51]. Smoking has also been associated with rotator cuff tears, as certain cigarette components can negatively influence muscle cell generation, apoptosis, and metabolism [52]. Finally, in older adults, the reduction in the microvessels in the tendon can lead to an increased susceptibility to fibrovascular hyperplasia, adiposis, atrophy, and calcification in rotator cuff tissue, which are conditions that are frequently associated with tears [53].

Therefore, future studies with larger sample sizes should not only focus on demographic and occupational variables but also incorporate metabolic and lifestyle factors to provide a more comprehensive understanding of the risk factors associated with epicondylosis. By doing so, we can develop more effective prevention and treatment strategies that address both the biomechanical and metabolic aspects of this condition.

### 4.1. Limitations and Strengths

This study is constrained by several limitations that merit consideration. Firstly, this research was confined to a specific geographic area, which complicates the generalizability of the findings. Future studies encompassing larger and more diverse populations across broader geographical regions are recommended to extrapolate the results to a broader context. Additionally, the data collection relied on one-time consultations and participant self-reporting, potentially introducing biases or inaccuracies. The precision of the clinical diagnoses of epicondylosis and the characterization of the variables may also be subject to limitations, given the potential variations in diagnostic practices among the physicians or the inclusive criteria. Controlling for confounding factors that could influence the likelihood of developing epicondylosis, such as the participants’ athletic and occupational activities, was challenging. Furthermore, survey-based data collection methods are susceptible to recall bias, despite our efforts to design clear and specific survey questions. This may have affected the accuracy of the reporting of past injuries or symptoms. Future studies employing more objective measures or longitudinal designs could mitigate these limitations and offer a more comprehensive understanding of the relationship between epicondylosis and adjacent joint injuries.

Despite these limitations, this study benefits from several strengths. The cross-sectional design enabled the simultaneous assessment of multiple variables, providing a snapshot of the association between epicondylosis and adjacent joint injuries. The inclusion of a diverse sample from multiple primary healthcare centers enhances the generalizability of the findings to similar populations. Furthermore, the comprehensive data collection process, incorporating detailed demographic, clinical, and occupational variables, strengthens the depth of the analysis and the interpretation of the results. Lastly, our adherence to the ethical guidelines, including our compliance with the Declaration of Helsinki and data protection regulations, underscores this study’s integrity and ethical conduct.

### 4.2. Future Research

Given the results of this study, future observational research, with larger sample sizes, is needed to further investigate both sex and occupational differences in the prevalence of epicondylosis. In addition, clinical trials should be conducted with patients diagnosed with epicondylosis to compare the efficacy of localized treatments targeting the specific area of injury versus global therapeutic approaches that take into account the entire upper extremity. These studies could assess whether treating epicondylosis with consideration of the health of the adjacent joint produces better outcomes than treatments focused on the elbow joint alone.

This preliminary research serves as a starting point for future research on epicondylosis. The findings and limitations identified provide a basis for further research. Researchers and healthcare professionals are encouraged to use these results to design comprehensive studies to further explore the epidemiology, risk factors, diagnosis, and treatment of epicondylosis. The observed association of previous injuries with epicondylosis highlights the potential importance of taking previous musculoskeletal conditions into account when understanding and treating this disorder.

## 5. Conclusions

In this study, lateral tendinopathy emerged as the most prevalent form of elbow tendon injury observed, followed by medial and bilateral cases. The right upper limb was notably more affected than the left, which is consistent with its dominant usage in daily activities. A significant proportion of the participants reported prior musculoskeletal injuries in the affected upper limb, which were primarily musculotendinous in nature. However, this study’s findings regarding the association between previous injuries and these tendon pathologies should be interpreted cautiously due to the potential biases, such as this study’s limited geographical scope and its reliance on self-reported data.

These preliminary insights underscore the need for larger-scale observational studies and clinical trials to further elucidate the relationship between previous injuries to an adjacent joint and the development of these tendon conditions. Addressing these research gaps will contribute to a more comprehensive understanding of risk factors, thereby informing more effective prevention and treatment strategies in clinical practice.

## Figures and Tables

**Table 1:** Association between type of injury and other variables.

		Lateral Epicondylosis	Medial Epicondylosis	Bilateral Epicondylosis *	Sig
	male	39/90 (43.4%)	3/90 (3.3%)	2/90 (2.2%)	
Sex	female	28/90 (31.1%)	9/90 (10.0%)	9/90 (10%)	0.01
	right	59/90 (65.5%)	11/90 (12.2)	9/90 (10.0%)	
Dominant upper limb	left	8/90 (8.8%)	1/90 (1.1%)	2/90 (2.2%)	0.764
	right	47/90 (52.2%)	6/90 (6.6%)	5/90 (5.5%)	
	left	16/90 (17.7%)	4/90 (4.4%)	5/90 (5.5%)	
Injured upper limb	both	4/90 (4.4%)	2/90 (2.2%)	1/90 (1.1%)	0.33
	occupational	47/90 (52.2%)	8/90 (8.8%)	3/90 (3.3%)	0.022
Cause of occurrence	non-occupational	20/90 (22.2%)	4/90 (4.4%)	8/90 (8.8%)	
	yes	12/90 (13.3%)	2/90 (2.2%)	2/90 (2.2%)	
	no	51/90 (56.7%)	8/90 (8.9%)	8/90 (8.9%)	
Previous existence of adjacent joint lesions in unaffected upper limb	don’t know	4/90 (4.4%)	2 (2.2%)	1/90 (1.1%)	0.798
	yes	56/90 (62.2%)	9/90 (10%)	7/90 (7.7%)	
Manual loading by occupation	no	11/90 (12.2%)	3/90 (3.3%)	4/90 (4.4%)	0.227
Time of evolution (days)		247.9 (217.3)/90 (100%)	183.8 (130.7)/90 (100%)	600 (1461.9)/90 (100%)	0.39

* The previous existence of lesions in the adjacent joints in the unaffected upper limb, where the data reflects the responses of those with bilateral involvement. For the cases of bilateral epicondylosis, this column indicates the existence of previous lesions in the unaffected limb at the time of the survey. Therefore, the data reflect lesions in the other upper limb that was unaffected at the time of the survey and should not be interpreted as the absence of bilateral involvement.

**Table 2:** Association between injured upper limb and other variables.

		Injured Upper Limb			
		Left	Right	Both	Sig
	male	13/90 (14.4%)	28/90 (31.1%)	3/90 (3.3%)	
Sex	female	12/90 (13.3%)	30/90 (33.3%)	4/90 (4.4%)	0.901
	right	18/90 (20%)	55/90 (61.1%)	6/90 (6.6%)	
Dominant upper limb	left	7/90 (7.7%)	3/90 (3.3%)	1/90 (1.1%)	0.014
	occupational	16/90 (17.7%)	36/90 (40%)	1/90 (1.1%)	0.466
Cause of occurrence	non-occupational	99/90 (10%)	22/90 (24.4%)	0/90 (0%)	
	yes	12/90 (13.3%)	25/90 (27.7%)	4/90 (4.4%)	0.915
Previous existence of adjacent joint lesions in unaffected upper limb	no	13/90 (14.4%)	33/90 (36.6%)	3/90 (3.3%)	
	yes	21/90 (23.3%)	44/90 (48.8%)	7/90 (7.7%)	
Manual loading by occupation	no	4/90 (4.4%)	14/90 (15.5%)	4/90 (4.4%)	0.27
Time of evolution (days)		393.8 (980.8)/90 (100%)	226.2 (193.5)/90 (100%)	350 (197.6)/90 (100%)	0.104
Age		47.5 (11.2)/90 (100%)	44.7 (12.1)/90 (100%)	46.1 (5.8)/90 (100%)	0.59

## Data Availability

Data are available upon request.
